# Measuring Vaccine Confidence: Introducing a Global Vaccine Confidence Index

**DOI:** 10.1371/currents.outbreaks.ce0f6177bc97332602a8e3fe7d7f7cc4

**Published:** 2015-02-25

**Authors:** Heidi J Larson, William S Schulz, Joseph D Tucker, David M D Smith

**Affiliations:** Department of Infectious Disease Epidemiology, London School of Hygiene and Tropical Medicine, London, UK; Department of Infectious Disease Epidemiology, London School of Hygiene & Tropical Medicine, London, UK; University of North Carolina at Chapel Hill (UNC) Medical School and UNC Project-China, University of North Carolina, Chapel Hill, North Carolina, USA; Department of Infectious Disease Epidemiology, London School of Hygiene & Tropical Medicine, London, UK

**Keywords:** vaccine hesitancy

## Abstract

Background. Public confidence in vaccination is vital to the success of immunisation programmes worldwide. Understanding the dynamics of vaccine confidence is therefore of great importance for global public health. Few published studies permit global comparisons of vaccination sentiments and behaviours against a common metric. This article presents the findings of a multi-country survey of confidence in vaccines and immunisation programmes in Georgia, India, Nigeria, Pakistan, and the United Kingdom (UK) – these being the first results of a larger project to map vaccine confidence globally. Methods. Data were collected from a sample of the general population and from those with children under 5 years old against a core set of confidence questions. All surveys were conducted in the relevant local-language in Georgia, India, Nigeria, Pakistan, and the UK. We examine confidence in immunisation programmes as compared to confidence in other government health services, the relationships between confidence in the system and levels of vaccine hesitancy, reasons for vaccine hesitancy, ultimate vaccination decisions, and their variation based on country contexts and demographic factors. Results. The numbers of respondents by country were: Georgia (n=1000); India (n=1259); Pakistan (n=2609); UK (n=2055); Nigerian households (n=12554); and Nigerian health providers (n=1272). The UK respondents with children under five years of age were more likely to hesitate to vaccinate, compared to other countries. Confidence in immunisation programmes was more closely associated with confidence in the broader health system in the UK (Spearman’s ρ=0.5990), compared to Nigeria (ρ=0.5477), Pakistan (ρ=0.4491), and India (ρ=0.4240), all of which ranked confidence in immunisation programmes higher than confidence in the broader health system. Georgia had the highest rate of vaccine refusals (6 %) among those who reported initial hesitation. In all other countries surveyed most respondents who reported hesitating to vaccinate went on to receive the vaccine except in Kano state, Nigeria, where the percentage of those who ultimately refused vaccination after initially hesitating was as high as 76%) Reported reasons for hesitancy in all countries were classified under the domains of “confidence,” “convenience,” or “complacency,” and confidence issues were found to be the primary driver of hesitancy in all countries surveyed.

## Related Articles

The article is part of the *PLOS Currents Outbreaks *"Vaccine Hesitancy Collection".

## Background

Understanding the dynamics of vaccine confidence is of great importance for global public health. Few published studies permit global comparisons of vaccination sentiments and behaviours against a common metric. To help address this knowledge gap, this article presents the findings of a multi-country survey of confidence in vaccines and vaccination programmes in Georgia, India, Pakistan, Nigeria, and the United Kingdom (UK) – these being the first results of a larger project to map vaccine confidence globally.

While more detailed local studies are important to inform appropriate interventions, we have observed significant global dynamics that influence vaccine confidence and the spread of vaccine sentiments. Studying these large-scale phenomena requires surveys at an international scale. The intention of our current global mapping effort, therefore, is not to distract from invaluable local details, but rather to pull back the lens of observation, so that signals of change can be detected and responded to as appropriate, and trends can be identified and studied in relation to each other, so that global dynamics can be discovered and understood.

***Defining Vaccine Confidence***

Public confidence in vaccines is, above all, a phenomenon of public trust. Fittingly, the Oxford English Dictionary defines “confidence” as “the mental attitude of trusting in or relying on a person or thing”.^1^ In the context of vaccination, confidence implies trust in the vaccine (the product), trust in the vaccinator or other health professional (the provider), and trust in those who make the decisions about vaccine provision (the policy-maker).

These trusting relationships are important because, in accepting vaccination, the public relies on the integrity, competence, and good faith of public health and government authorities to recommend vaccines appropriately, of private-sector actors to manufacture effective and uncontaminated products, and of health providers to administer them safely. The definition of trust as the "optimistic acceptance of a vulnerable situation in which the trustor believes the trustee will care for the trustor’s interest"^2^ is relevant here. Both trust and confidence are important for understanding perceptions of vaccines. Trust fundamentally depends on perceptions of competence and motive.^3^ Importantly, trust and confidence in vaccines are dynamic and contextual and depend on perceptions of competence and motive of the provider—both vaccine producers as well as health professionals—as well as the politicians who determine the policies.

There are many related terms used in the vaccine confidence literature,^4^^,^ 5 ^,^ 6 ^,^ 7 which has increasingly looked at vaccine hesitancy as a possible indicator of waning confidence. In March 2012, the WHO Strategic Advisory Group of Experts (SAGE) on Immunisation convened a Working Group^8^ in recognition of the growing prevalence of vaccine questioning and hesitation, which sometimes lead to vaccine delays and refusals. In the context of the working group research and deliberations, three key domains of influence driving vaccine hesitancy were defined: confidence (trust in the safety or efficacy of the vaccine), convenience (ease of access), and complacency (perception of the risk of disease and importance of immunisation).^9^ In the research addressed in the paper, we examine overall confidence in the health system generally as well as immunisation, then investigate vaccine hesitancy and its reasons, and finally query whether hesitancy led to acceptance or refusal of a vaccine or vaccines. We categorise the reasons for hesitancy reported into the domains of confidence, convenience, and complacency.

Individuals may lack confidence in the safety or efficacy of vaccines for a variety of reasons. They may lack confidence as a result of negative experiences with the product, providers, or those making the policy decisions. They may hold religious or philosophical beliefs that lead them to prefer traditional rites, prayers, or homeopathic remedies over biomedical interventions.

Vaccine confidence is not merely an individual phenomenon, but a social and political phenomenon as well. When vaccine-hesitant individuals reach a critical mass in a population, and do not receive adequate attention and engagement from health authorities on the specific issues they may have with a vaccine, they may form coalitions of varying looseness or consensus. Examples include coalitions^10^ which pressured the Indian government to suspend an HPV vaccine demonstration project, and another which pressured the suspension of the HPV vaccine recommendation in Japan.

Vaccine confidence metrics can provide valuable cues to changing public sentiment about vaccines and the potential for consequent changes in vaccine coverage.. More refined studies can provide needed local detail to understand the drivers of shifts in confidence and inform the appropriate response needed.

Measuring vaccine confidence is an emerging science. In developing our Vaccine Confidence Index (VCI), we have taken cues from other social science tools that measure confidence more generally. The closest analogue to the VCI is the Consumer Confidence Index (CCI), which measures consumer confidence, defined as the degree of optimism about the state of the economy – deemed important because consumers’ confidence is reflected in their spending and saving behaviour, which in turn impacts the larger economy. . The CCI is dependent on larger social, national, and regional economic issues. The Vaccine Confidence Index (VCI) is analogous to the CCI in that it too places a finger on the pulse of a set of public sentiments, which influence vaccination behaviours, with consequences for the whole population. In the case of the VCI, the sentiments in question are confidence in vaccination and the entities with which it is associated, and, like the sentiments measured by the CCI, vaccine sentiments are influenced by broader social dynamics.

The VCI can likewise be a potentially useful tool for researchers and policy-makers, and could provide an empirical basis for monitoring vaccine confidence over time in a number of regions. We report here the results from the first five countries surveyed as an initial pilot of a Vaccine Confidence Index. These findings indicate the viability of this approach to measure vaccine-related confidence (that is, sentiments as they influence vaccination behaviours), and illustrate the relationships between these sentiments and public attitudes towards health services more broadly. These confidence metrics are currently being rolled out in additional countries, contributing to our global mapping of vaccine confidence, which will be updated over time and strengthened with more local level confidence mapping.

***Country Backgrounds***

The five countries chosen for the initial launch of the Vaccine Confidence Index have each faced a confidence crisis, and they have addressed these confidence challenges with differing levels of success.

Nigeria was the site of one of the most significant episodes of a vaccine confidence crisis that had substantial public health consequences. In August 2003, a polio vaccination boycott was announced in five northern states and persisted in Kano State for eleven months, only being resolved in July 2004. The boycott seeded a resurgence of polio in Nigeria as well as outbreaks across three continents (Figure 1),^11^ and cost over $500 million.^12^ Poliovirus incidence peaked in 2006, with 1143 confirmed cases, but has since dropped back down, now at its lowest-ever levels, with only 6 confirmed cases of wild polio virus reported at the end of 2014.^13^

**"A Warning from History":**
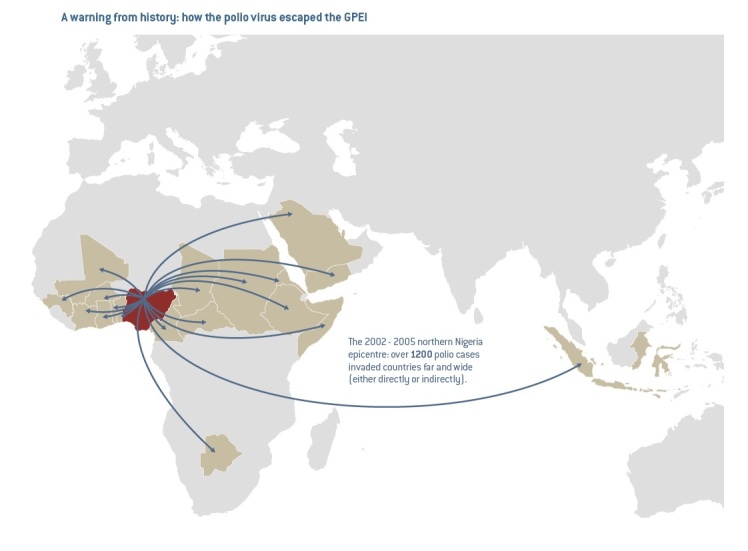
Published originally in the November 2012 Report of the Independent Monitoring Board of the Global Polio Eradication Initiative, this figure presents the spread of poliovirus from Nigeria following the 2003-2004 boycott.

As the rumours were building up in northern Nigeria just over a decade ago, the India polio programme also encountered distrust, including similar rumours of sterilisation, among marginalised and underserved communities in the states of Uttar Pradesh and Bihar. Years of targeted efforts to build trust and confidence, in the vaccine as well as in the polio programme as an institution, were key contributors to India’s being declared polio free a decade later in January 2014.

In 1998, the UK was the epicentre of perhaps the most infamous confidence crisis in recent memory, which led to widespread anxieties, declines in vaccine acceptance and consequent measles outbreaks, following the publication of now-debunked research by Andrew Wakefield^14^ suggesting links between the MMR vaccine and autism. MMR vaccine coverage reached a nadir in 2003 in England, and it took years of routine opinion surveys to better understand the nature of public concerns, followed by community engagement and trust building, before the MMR vaccine coverage rate finally returned to pre-1998 levels in 2014, 15 years after the publication which prompted the public panic.

In 2002, Georgia experienced a suspected adverse event following a Hepatitis B vaccination, which prompted negative media and public anxiety. While confidence levels and Hepatitis B vaccine acceptance have improved following the initial decline, they have still not reached pre-2002 levels.^15^

In Pakistan, a ban on polio vaccination in North and South Waziristan has persisted since June 2012 linked to a demand to stop Drone strikes. 16 ^,^ 17 ^,^ 18 Not only has polio vaccination become highly politicised, but the ban in Waziristan has fuelled both local and international polio outbreaks – including an outbreak of the Pakistani strain in Syria, already burdened by a civil war and broken health systems. Pakistan’s own confirmed polio cases jumped from 74 in 2012 to 193 in 2013, and then to 305 in 2014, and the total number of Wild polio virus (WPV1) in neighboring Afghanistan doubled from 14 cases in 2013 to 28 cases in 2014, largely due to cross border transmission from Pakistan.^19^

## Methods

The generation of data on vaccine confidence reported and analysed here was made possible through an agreement between The Vaccine Confidence Project at the London School of Hygiene & Tropical Medicine and “Global Public Health Polling Network” jointly managed by ORB International, UK and Gallup Pakistan, affiliates of WIN-Gallup International. The Global Public Health Polling Network incorporated a set of questions on vaccine confidence, developed by the Vaccine Confidence Project, into larger surveys being conducted in the many countries in which WIN-Gallup International operates. The fieldwork was conducted by ORB International in the UK and (with the assistance of Dr. Ibrahim Yisa) in Nigeria, and by Gallup Pakistan, C Voters, and GORBI in Pakistan, India, and Georgia respectively. The resulting dataset offers not only a broad (and growing) international sample of vaccination sentiments and behaviours, but also includes extensive data on respondents’ social context and other attributes, collected as part of the larger surveys in these countries. Data collection methods in each of the five countries surveyed are described below.

Data collection in Pakistan consisted of face-to-face in-house interviews in Urdu with 2609 respondents, selected by multi-stage random area probability sampling, between 31 March and 7 April 2014, in Punjab, Sindh, Khyber Pakhtunkhwa, and Baluchistan. Findings were then weighted according to rural and urban population share in each province, based on the 1998 Population Census.

Data collection in the UK consisted of online interviews with 2055 respondents, between 23 and 24 April 2014, in England, Scotland, and Wales. To compensate for the effects of self-selection of respondents choosing to participate in the survey, findings were then weighted on demographic variables, according to census figures.

Data collection in India consisted of computer-assisted telephone interviewing (CATI) of 1259 respondents, selected from a random sample of phone numbers covering all regions of India, between 9 and 11 April 2014. Findings were then weighted according to the known census profile. Interviews were conducted in the relevant local languages: Hindi, Punjabi, Urdu, Gujarati, Marathi, Kannada, Malayalam, Tamil, Telugu, Odiya, Bangla and Asamiya.

In Nigeria, both households and health providers were surveyed. Data collection in Nigerian households consisted of face-to-face interviews (using personal digital assistants [PDAs]) with 12554 respondents from Enugu, Jigawa, Kaduna, Kano, and Lagos, selected from master sample frames for enumeration areas defined by the National Bureau of Statistics (NBS). Survey materials were translated into Yoruba, Igbo, and Hausa. Findings were then weighted according to information about these enumeration areas, also provided by the NBS.

A total of 1272 providers in Nigeria were also interviewed in the 968 facilities participating in the survey. Providers were defined as health workers trained to provide obstetric care and child care services, on the assumption that these individuals have final responsibility for obstetric and child health care.

Data collection in Georgia consisted of computer assisted personal interviewing (CAPI) of 1000 respondents, selected using multi-stage stratified sampling based on quotas for age, gender, and education, carried out between 23 August and 1 September 2014. Survey materials were translated in to Georgian and Russian.

In our analysis, for respondents without children under five, only a general question was asked about perceptions of vaccine coverage. (see Figure 6, appendix) More detailed questions focused on the subset of respondents who were parents of children under 5 (except in Georgia, where parents were defined as having children under 15). We examine relationships between vaccination behaviour and opinions on vaccination and government health services more broadly, reported instances of vaccine hesitancy and their reasons, ultimate decision about whether to vaccinate, and variation in responses based on country contexts and demographic factors. Reasons for hesitancy given by vaccine-hesitant respondents were classified as relating to confidence (concerns about the safety or efficacy of the vaccine, previous bad experiences, or preference for alternative health approaches), convenience (access issues), complacency (perceptions that the vaccine was unimportant or unnecessary), or other responses categorised as “other/don’t know/no reason.”

## Results

***Overview***

The numbers of respondents in each country were: India (n=1259); Pakistan (n=2609); UK (n=2055); Nigeria Households (n=12554); Nigeria Providers (n=1272), Georgia (n=1000).

Figure 2 shows a breakdown of respondents in each country by whether they had children under five, if so, whether they had ever hesitated to vaccinate their child, and if so, whether they ultimately had the vaccine or did not have the vaccine. Georgia shows the highest percentage (60%) of vaccine refusers among those who reported hesitancy, followed by Nigeria Households where 22.7% of households reporting hesitancy refused vaccination.

**Table 1: Survey Size and Prevalence of Hesitancy and Refusal:** Vaccination behaviours of hesitancy and refusal are presented both in absolute numbers, and as proportions. Hesitancy is presented as a proportion of respondents with children equal to or under five (RCU5), except for Georgia(*) which represents under 15 years of age, and refusal is presented as a proportion of respondents who hesitated.

	Survey size	With child ≤5 years old (RCU5)	Hesitants	Hesitants as % of RCU5s	Outright refusers	Outright refusers as % of hesitants
India	1259	288	36	12.5%	6	16.7%
Pakistan	2609	709	99	13.9%	15	15.2%
UK	2055	196	48	24.5%	13	27.1%
Nigeria Households	12554	3687	308	8.4%	70	22.7%
Nigeria Provider	1272	519	44	8.5%	5	11.4%
Georgia	1000	474*	35	7.4%	21	60%

The UK sample contained fewer respondents with children under five years of age (RCU5s) than the other countries surveyed. UK RCU5s were more likely to hesitate to vaccinate, compared to RCU5s in other countries. In Georgia, by contrast, hesitants made up a smaller proportion of RCU5s, but of those who hesitated, a majority reported not receiving the vaccine. In all countries but India, RCU5s were more likely (compared to respondents who did not have children under five) to believe that all or most people in their community get their children vaccinated, and less likely to say they “don’t know” how many get their children vaccinated.

***Association with Confidence in Other Services***

In all five countries surveyed, overall confidence in immunisation was high; outside of the UK where confidence in emergency services was slightly higher than in immunisation programmes, confidence in immunisation services was higher than confidence in family planning services, in health workers and in the general health system (see Figure 2).

**Overview of Confidence Comparisons:**
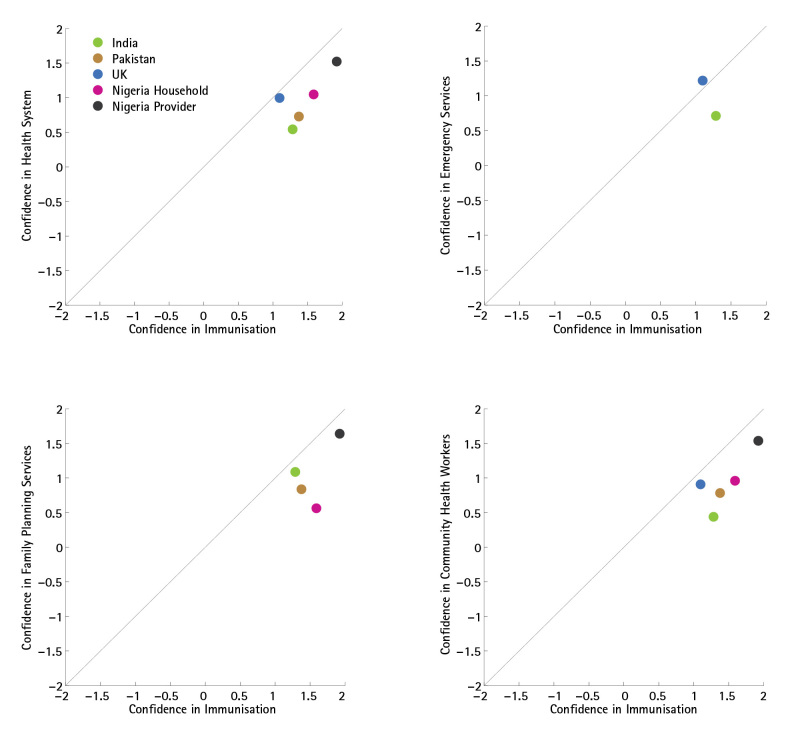
These plots compare confidence scores for immunisation programmes against confidence scores in the larger health system, in emergency services, in family planning services, and in community health workers, in each country for which the requisite data are available.

Public confidence in immunisation programmes was more closely associated with confidence in the broader health system in the UK (Spearman’s ρ=0.60), compared to Nigeria (ρ=0.55), Pakistan (ρ=0.45), and India (ρ=0.42), which all had higher confidence in immunisation services than in the health system generally. In all countries but the UK, immunisation services received stronger confidence ratings than the health system. Providers in Nigeria expressed very high confidence in both immunisation programmes and family planning services, and as a result health provider confidence in immunisation programmes in Nigeria showed a very strong association (ρ=0.68) with confidence in the health system (see Figure 3). Indeed, Nigerian health providers’ high confidence ratings for all services resulted in this group of respondents showing the strongest associations between confidence in immunisation programme and confidence in all other services (compared to the confidence expressed by the general public in any of the countries surveyed).

**Confidence in Immunisation Programme and Health System:**
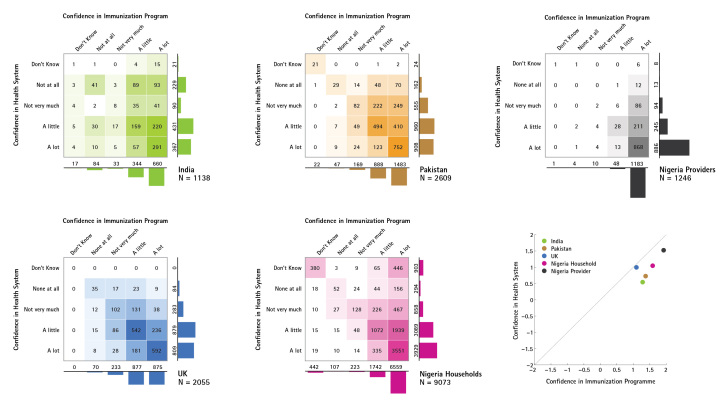
These contingency tables illustrate the varying association between confidence in immunisation programmes and confidence in the broader health system in India, Pakistan, the UK, and households and providers in Nigeria.

Public confidence in immunisation programmes was more closely associated with confidence in emergency services in the UK (ρ=0.55), compared to India (ρ=0.45). In India, confidence in immunisation services exceeded confidence in emergency services, whereas in the UK emergency services received slightly greater confidence than immunisation services (see Figure 7, Appendix).

Public confidence in immunisation programmes was more closely associated with confidence in family planning services in India (ρ=0.58) and Pakistan (ρ=0.56) than in Nigeria (ρ=0.43), where lower confidence in family planning (average confidence score of 0.5) persisted among those with high confidence in immunisation (average confidence score of 1.6). Among Nigerian providers, the association between confidence in immunisation programmes and confidence in family planning services was very high (ρ=0.76). Confidence in immunisation programmes was stronger than confidence in family planning services in all countries (see Figure 8, Appendix).

Public confidence in immunisation programmes was more closely associated with confidence in community health workers in the UK (ρ=0.58) than in Pakistan (ρ=0.48), Nigeria (ρ=0.53), or India (ρ=0.36), and in all countries confidence was higher in immunisation services than in community health workers. As in the other comparisons described above, Nigerian providers expressed high confidence both in immunisation programmes and community health workers, leading to a strong association between confidence ratings for each (ρ=0.67). Confidence in immunisation programmes was stronger than confidence in community health workers in all countries, though only by a small margin for the UK public. (See Figure 9, Appendix).

***Confidence as a Sentiment Linked to Behaviours of Hesitancy and Refusal***

Since vaccine confidence has been defined here as a sentiment or mental attitude that increases the likelihood of hesitating and/or refusing to vaccinate, it is important to ask how this relationship is reflected in these data. Figure 4 illustrates, in each country for which the requisite data are presently available, the variation in the probability of vaccine hesitancy among RCU5s, depending on reported level of confidence in immunisation programmes. In every country, we observe a clear trend in which lower levels of confidence are associated with higher levels of hesitancy. There is considerable variation between countries in the probability of hesitancy at a given confidence level. For example, reporting no confidence at all in immunisation programmes is associated with a 17% hesitancy rate in India, compared to 50% in Pakistan. It should be noted that reported confidence in immunisation programmes is merely the most obvious single variable in this dataset to interpret as “vaccine confidence,” and other variables and combinations of variables would naturally be incorporated into a refined “Vaccine Confidence Index” metric.

**Relationship between Vaccine Confidence and Vaccination Behavior:**
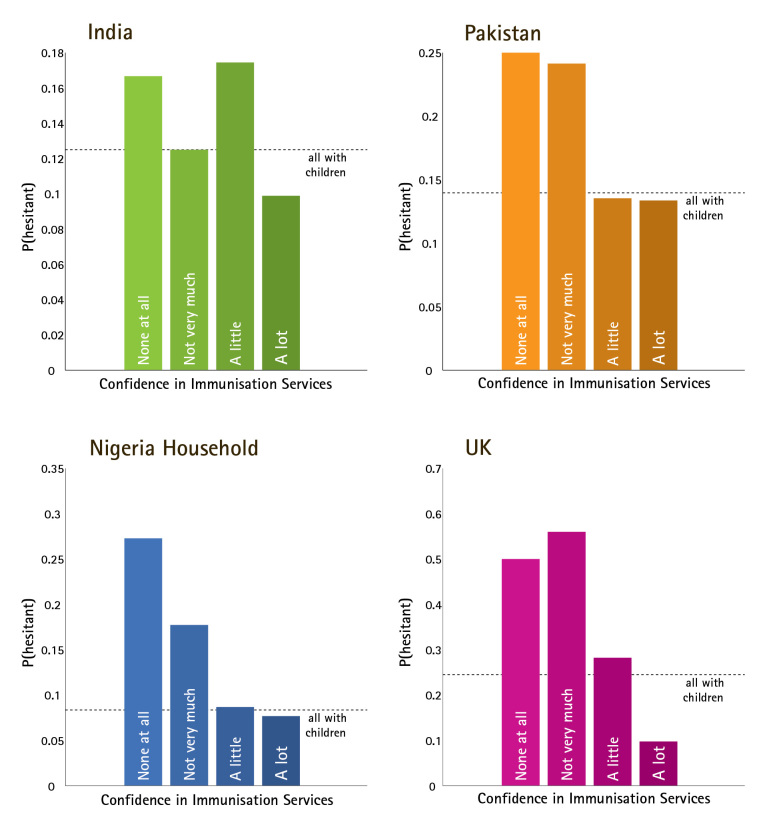
Probability of having hesitated to vaccinate in the past, according to expressed level of confidence in immunisation programmes, in each country for which the requisite data are available. Dotted lines indicate average hesitancy rate for RCU5s, irrespective of confidence in immunisation programmes.

***Reasons for Hesitancy***

Reasons for hesitancy given by vaccine-hesitant respondents were classified as relating to confidence, convenience, complacency, or other/don’t know (DK)/no reason (NR). Overall, the highest percentage of reasons for hesitancy was due to confidence issues. Figure 5 shows the distribution of hesitant respondents in each country, categorised by their reason for hesitancy (confidence, convenience, complacency, or other), and by ultimate behavioural outcome (vaccine acceptance or refusal).

**Table 2: Reasons for Hesitancy:** Reasons for hesitancy were classified using the categories of confidence, convenience, and complacency.

	Confidence	Convenience	Complacency	Other/DK/NR
Georgia	69%	6%	8%	17%
India	49%	18%	3%	31%
Nigeria	36%	20%	18%	26%
Pakistan	33%	20%	6%	41%
United Kingdom	79%	6%	13%	1%

**Reasons for Hesitancy:**
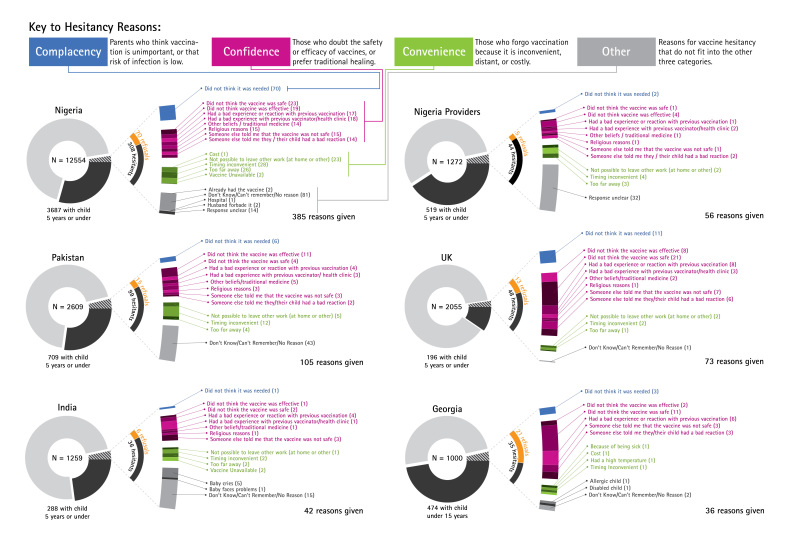
These graphs illustrate the total sample size (whole circle), composed of respondents who were not parents of children under 5 (light grey slice), parents who never hesitated to vaccinate their children (medium grey slice), and hesitant parents (dark grey slice). The curved grey-and-orange dial to the right of the hesitant slice shows the proportion of hesitant parents who ultimately refused the vaccine (orange), and those who eventually went on to get the vaccine (grey). Hesitant parents are further disaggregated according to the reasons they gave for their hesitation, which are grouped into complacency (blue), confidence (magenta), convenience (green), and other (grey).

***Key Findings at the Nigerian State Level***

Focusing on findings from the Nigerian states of Enugu, Jigawa, Kaduna, Kano, and Lagos, it is still possible to see the effects of the 2003-2004 boycott, ten years on. In Kano state, the site of the longest-lasting boycott, hesitancy rates are not exceptionally high, but unlike in other states, a high percentage (74%) of hesitants went on to refuse vaccination (see Table 3). These preliminary findings indicate some variation in “obstinacy” (tendency of hesitants to ultimately refuse), particularly evident in Kano and Enugu states.

**Table 3: Hesitancy and Refusal Rates at Nigerian State Level:** Hesitancy is given in absolute numbers and as a proportion of respondents with children under five years of age. Refusal is given in absolute numbers and as a proportion of hesitants.

State	With Child £ 5 yrs (RCU5)	Hesitants	Hesitants as % of RCU5s	Refusers	Refusers as % of Hesitants
Enugu	841	44	5.23 %	13	29.55 %
Jigawa	637	101	15.86 %	10	9.90 %
Kaduna	701	96	13.69 %	16	16.67 %
Kano	604	31	5.13 %	23	74.19 %
Lagos	904	36	3.98 %	8	22.22 %
Total	3687	308	8.35 %	70	22.73 %

## Limitations

There are a number of limitations in this first of a series of country vaccine confidence studies. First, survey methods varied somewhat between countries. For example, in Nigeria data were collected by face-to-face interviews conducted within households, while in the UK the survey was completed online. These different formats could have impacted responses.

Although the same core questions were repeated across the five countries, the full set of questions posed in the survey was not precisely the same in every country. This limited the breadth of comparisons possible, where, for example, surveys in India and the UK asked respondents about their confidence in emergency services, but other countries did not. Furthermore, in Georgia the local team did not include the questions on confidence in immunisation programmes or other health services so this phenomenon could not be considered in the comparison. Also respondents in Georgia were asked whether they had children under 15 years of age, as opposed to five years of age as in other countries surveyed.

These factors might introduce unaccounted-for variation in findings between countries. Therefore comparisons between country contexts should be made with some reserve, at least with these early results. Nonetheless, one of the striking results of these surveys is the remarkable consistency in trends observed across most or all countries, as discussed further below in the conclusions section.

## Conclusion

The first conclusion to draw from these findings is that medium-to-high confidence in vaccines and immunisation programmes is the norm, and vaccine hesitancy and refusals are relatively rare. Nonetheless, even small groups of hesitant or refusing individuals can severely undermine an immunisation programme in certain circumstances, such as when political actors in Nigeria and Pakistan mobilised local boycotts that have had both national and international repercussions. This begs the question, “How much confidence is enough?”

Second, the finding that higher confidence in immunisation programmes correlates with lower vaccine hesitancy and lends support to the premise that confidence in vaccination is connected to confidence in the broader system with which it is associated.

Thirdly, confidence issues constituted the most prevalent reasons for vaccine hesitancy and refusals (except in Georgia). Although the survey questions were designed by those within The Vaccine Confidence Project, and those coding free-form “other” responses were not blinded, the questions allowed ample opportunity for respondents to give answers other than those related to confidence, and the classification of answers within the confidence/ convenience/ complacency framework was agreed by independent coders.

Returning to the question of "how much confidence is enough?" there is no clear watershed confidence level that is consistent across every country – in India and the UK, hesitancy rises sharply between “a lot” and “a little” confidence, whereas in Pakistani and Nigerian households the distinction between “a little” and “not very much” appears to have more impact on behaviour. Linguistic differences between these countries may result in different translated meanings of “a little” and “not very much”. Alternatively, it is possible that contextual or demographic variables mediate between confidence sentiments and vaccination behaviour, and variation in these mediators gives rise to the between-country variations observed here.

At the societal level, the question of, “how much confidence is enough?” can be posed in terms of a “tipping point.” In other words, is there a critical proportion of the population that must remain vaccine-confident for the system as a whole to remain resilient to a “crisis of confidence,” in which doubt becomes prevalent enough that it becomes self-reinforcing? Is there a crucial point beyond which previously-confident laypersons begin questioning the vaccine, healthcare providers become less willing to promote it, and policy-makers consider withdrawing a recommendation for an effective and safe vaccine for fear of public disapproval? Is it more dangerous if a small part of the population to lose a great deal of confidence, or if a larger group becomes only slightly less confident? And, again, what contextual factors heighten the risk of a crisis at any given level of vaccine confidence?

Answering these questions will require data gathered from multiple countries, over time, which redoubles the need for confidence surveys at the global scale. This global vaccine confidence mapping initiative is the beginning of a longer-term effort, which will be refined and expanded to multiple countries. In effect, we are attempting to launch a large cohort study of as many countries of the world as possible. If the cohort is large enough, and the timescale is long enough, then it will become possible to relate “incident cases” of vaccine confidence crisis to the “exposures” measured through surveys of confidence and relevant contextual and demographic factors, permitting ascertainment of the “risk factors” for crises with both quantitative rigour and qualitative depth.

We expect that this Vaccine Confidence Index and the insights it generates will help inform the strengthening of local and global vaccine confidence in the years to come.

## Competing Interests

The authors have declared that no competing interests exist.

## Correspondence

Heidi.Larson@lshtm.ac.uk

## Figures and Tables

**Figure:**
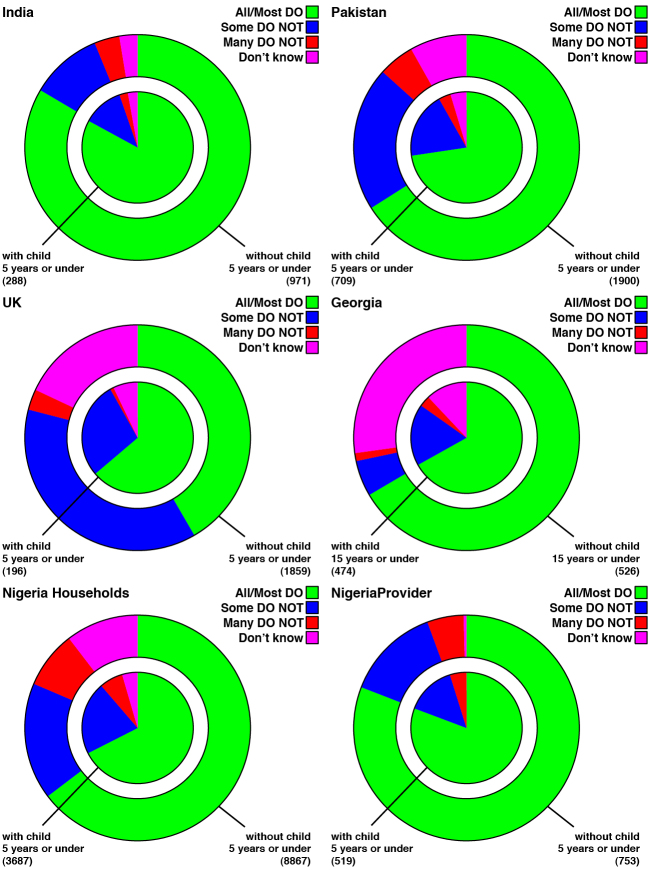
**Figure 6: Perceptions of vaccine coverage ** This figure compares perceptions of how many people get vaccinated among those respondents without children under 5 (outer ring) to those reported by those with children under five RCU5s (inner circle).

**Figure:**
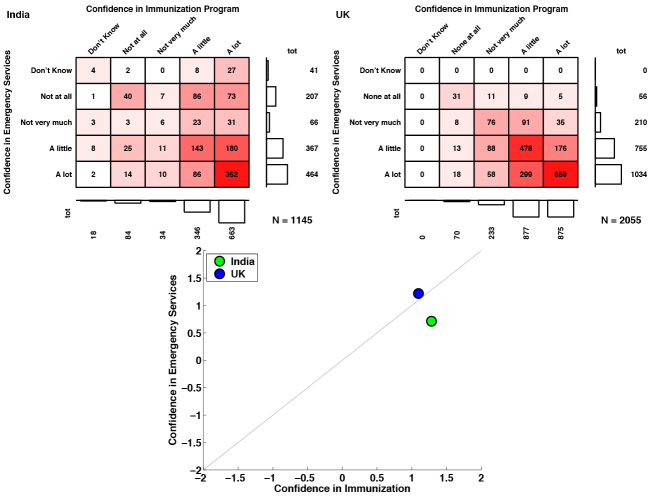
**Fig. 7: Confidence in Immunisation Programme and Emergency Services** These contingency tables illustrate the varying association between confidence in immunisation programmes and confidence in emergency services in India and the UK.

**Figure:**
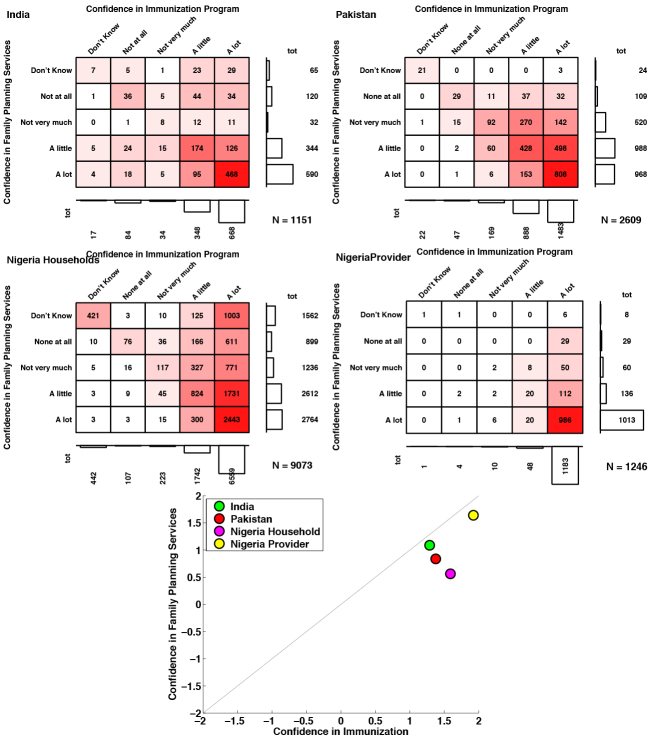
**Fig. 8: Confidence in Immunisation Programme and Family Planning** These contingency tables illustrate the varying association between confidence in immunisation programmes and confidence in family planning programmes in India, Pakistan, and households and providers in Nigeria.

**Figure:**
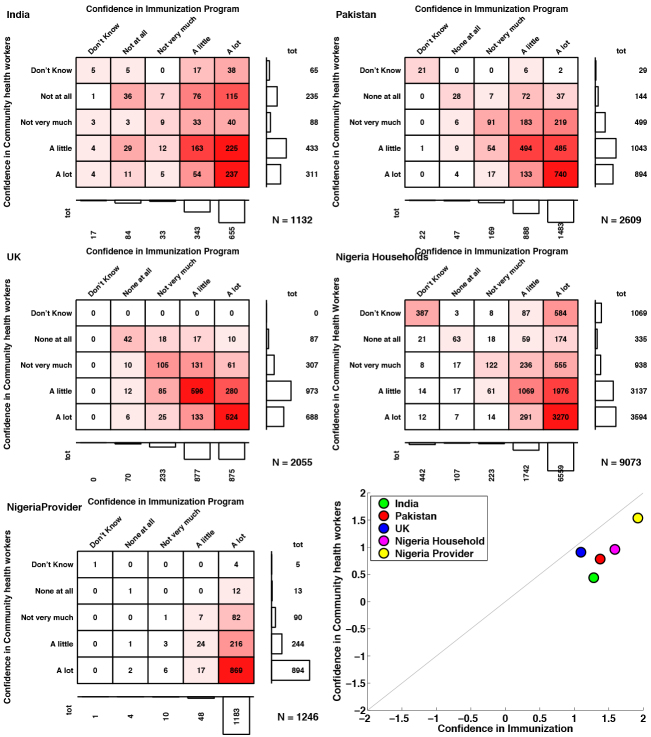
**Fig. 9: Confidence in Immunisation Programme and Community Health Workers** These contingency tables illustrate the varying association between confidence in immunisation programmes and confidence in community health workers in India, Pakistan, the UK, and households and providers in Nigeria.
